# Verrucoid Variant of Invasive Squamous Cell Carcinoma in Oral Submucous Fibrosis: A Clinicopathological Challenge

**DOI:** 10.7759/cureus.862

**Published:** 2016-11-04

**Authors:** Priya Ramani, C. Krithika, R. Ananthalakshmi, Mamta Singaram, Praveena Jagdish, Sunitha Janardhanan, Sathiyajeeva Jeevakarunyam

**Affiliations:** 1 Department of Oral Medicine and Radiology, Thai Moogambigai Dental College and Hospital; 2 Department of Oral Pathology, Thai Moogambigai Dental College and Hospital

**Keywords:** verrucous carcinoma, invasive squamous cell carcinoma, oral submucous fibrosis, serial sectioning, periodic acid schiff (pas), dysplasia, exophytic growth

## Abstract

Verrucous carcinoma (VC) is an exophytic, low-grade, well-differentiated variant of squamous cell carcinoma. It is described as a lesion appearing in the sixth or seventh decade of life that has minimal aggressive potential and, in long-standing cases, has been shown to transform into squamous cell carcinoma. Oral submucous fibrosis (OSMF) is a potentially malignant disorder, and about one-third of the affected population develop oral squamous cell carcinoma. The histopathological diagnosis of verrucous carcinoma is challenging, and the interpretation of early squamous cell carcinoma requires immense experience. Here we present a rare case of a 24-year-old male with OSMF transforming to verrucous carcinoma with invasive squamous cell carcinoma. Even though the case had a straightforward clinical diagnosis, the serial sectioning done for pathological diagnosis disclosed the squamous cell carcinoma.

## Introduction

VC is a highly differentiated variant of squamous cell carcinoma and is a very rare entity first described by Lauren V. Ackerman in 1948 [1]. VC is also known as Ackerman’s tumor, Buschke-Lowenstein tumor, oral florid papillomatosis, epithelioma cuniculatum, and carcinoma cuniculatum [1]. This tumor is slow-growing, locally invasive, unlikely to metastasize, and presents as a painless, exophytic, well-demarcated hyperkeratotic lesion resembling a cauliflower. Schrader, et al. described the spread of VC by lateral extension noting that VC is locally destructive but, if neglected, can invade the periosteum and bone [2]. It is more commonly seen in men than women, usually in the sixth and seventh decade of life [3]. The most common locations for occurrence in the oral cavity include buccal mucosa mandibular alveolar crest, gingiva, and tongue [4]. Tobacco in all forms, alcohol, and opportunistic human papillomavirus have been considered causative factors [5]. OSMF is a potentially malignant disorder that can transform into squamous cell carcinoma and, rarely, into VC. The coexistence of the squamous cell carcinoma with VC in OSMF is exceedingly rare. Herein, we report a case of a 24-year-old male with OSMF and VC with invasive squamous cell carcinoma.

## Case presentation

A 24-year-old male patient reported with a painless, white, solitary exophytic growth present on the left buccal mucosa for the past three months. The patient also had a complaint of a burning sensation for the past two years and difficulty in opening his mouth. He had a habit of chewing mawa (a mixture of areca nut pieces, tobacco, and slaked lime rubbed on polythene paper) for the past three years. He was using mawa four to five times daily, stacking it for 10 minutes in the left buccal mucosa and then spitting it out. He also had a history of occasionally consuming alcohol.

**Figure 1:**
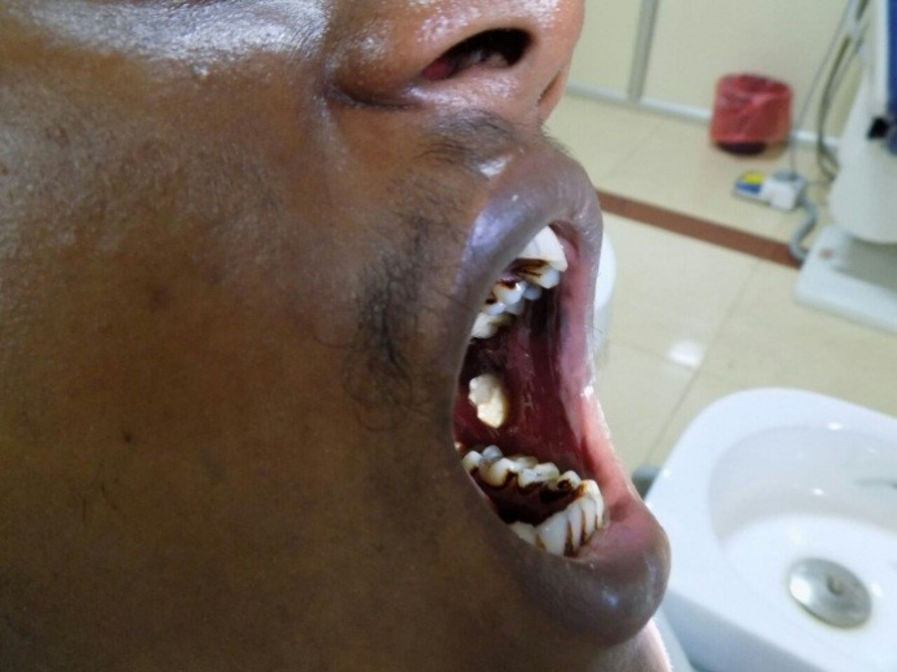
White papillary growth involving left buccal mucosa.

An intraoral examination revealed a single, whitish exophytic growth with a papillary projection measuring approximately 2 cm × 1 cm. The surface appeared to be corrugated and rough. The growth was present exactly on the occlusal line of the buccal mucosa. Anteriorly, it was 3 cm away from the left commissure of the lip and, posteriorly, 2 cm away from the retromolar area (Figure 1).

On palpation, the growth was tender and firm in consistency. Vertical fibrotic bands were palpable on the left retromolar area. A single left submandibular lymph node measuring approximately 1 cm × 1 cm was freely movable, firm in consistency and tender on palpation. Thus, based on the clinical findings, a provisional diagnosis of OSMF with VC of the left buccal mucosa was given. Considering the age of the patient, the size of the lesion, and its tendency to spread laterally, a complete excisional biopsy was performed. This tissue sample was subjected to histopathological analysis.

On macroscopic examination, the soft tissue specimen measured 2 cm × 1 cm and was firm with white papillary surface projections. The entire surface of the specimen was firm and grayish white in color. Both halves of the specimen were processed and stained with routine hematoxylin and eosin (H&E) stain. The histopathological picture revealed keratin plugging and wide, elongated rete ridges into the underlying fibrovascular stroma with mild dysplastic features. The infiltration of chronic inflammatory cells masked the basement membrane integrity, so the section underwent periodic acid-Schiff (PAS) and serial sectioning to examine the deeper tissues. Finally, a clinicopathological diagnosis of OSMF with early invasive squamous cell carcinoma from VC was made.

## Discussion

Oral cancer is rated as the second largest noncommunicable disease [6]. In India, the cancer mortality rate is expected to reach 0.70 million in 2026 [6]. Diagnosis of oral cancer from a preexisting, potentially malignant lesion requires a multidisciplinary approach. Two to 12% of oral cancer is verrucoid in nature [7]. The malignant transformation of OSMF requires a period of 17 years with a reported 7.6% transformation rate [8]. VC, which presents as a warty, thick keratotic lesion is usually asymptomatic or painless [4]. In case series studies done by Alkan, et al., lymph nodes were unaffected [9]. In our case, the patient was in his third decade of life with a small, painless growth on his buccal mucosa which was provoked by a short duration of mawa chewing and a single, firm and tender lymph node. Mawa is a combination of small pieces of areca nut, processed tobacco, and slaked lime rubbed on polythene paper for a stipulated time [10].

**Figure 2:**
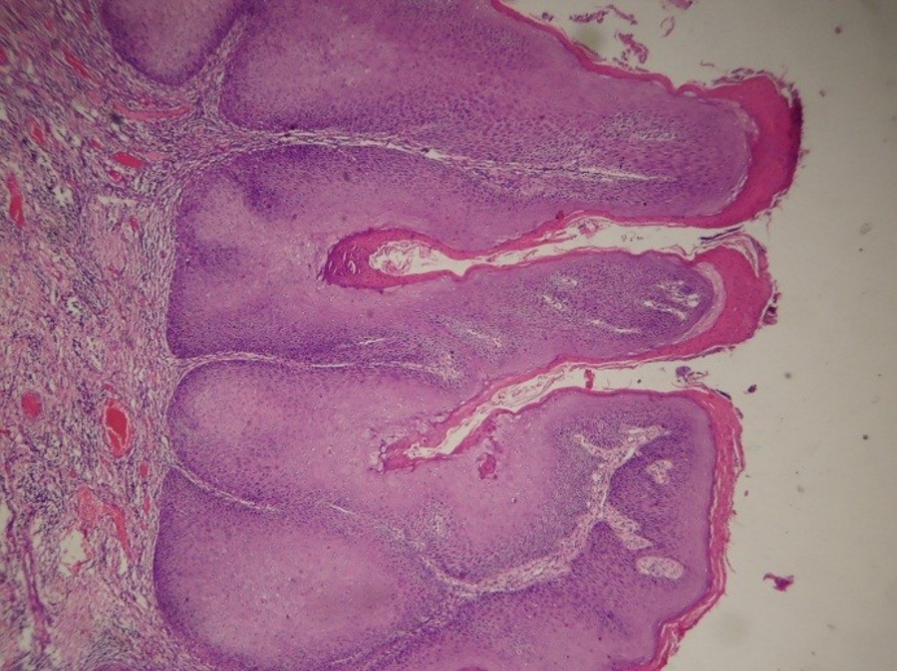
Photomicrograph of the HE-stained section shows a parakeratinized exophytic and endophytic growth with pushing borders, 10X, and endophytic growth with pushing borders, 10X.

**Figure 3:**
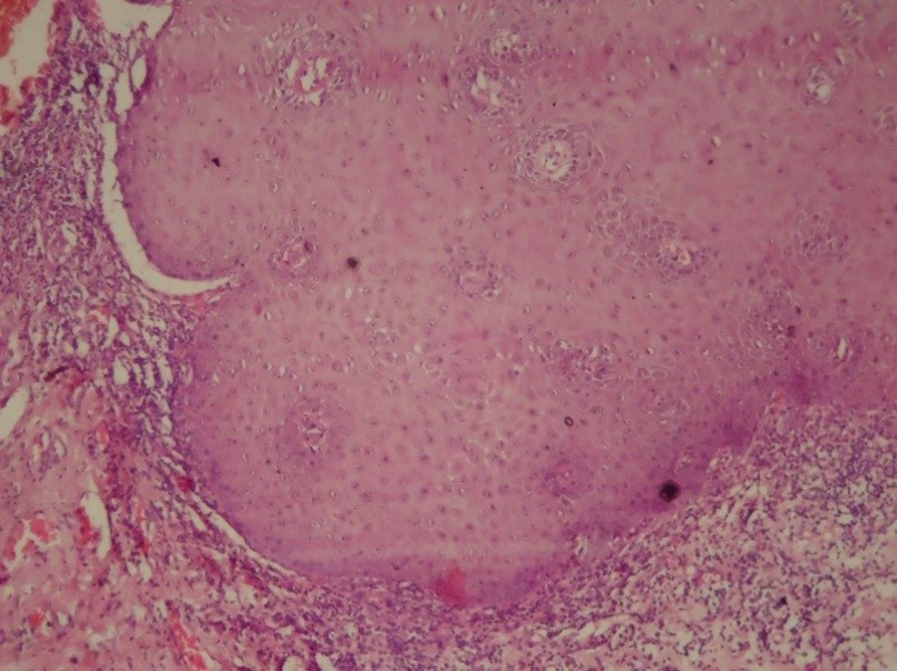
Dysplastic epithelium, HE stain, 40X.

Even with direct observation of VC features (Figure 2), along with mild dysplastic epithelial islands adjacent to the basement membrane, the initial histopathological picture was confusing (Figure 3).

**Figure 4:**
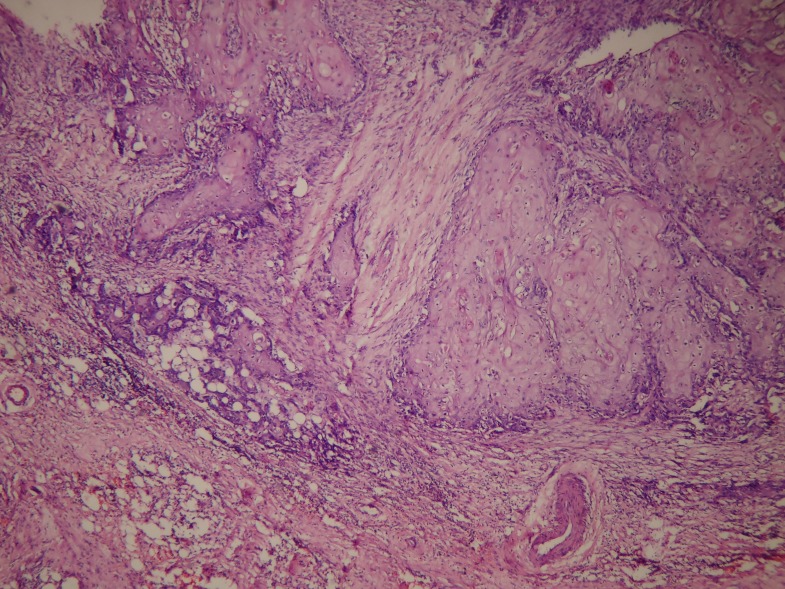
Squamous malignant epithelial islands in the connective tissue stroma, 10X.

With the masking of chronic inflammatory cell infiltration in the basement membrane, the specimen was processed for PAS. The PAS did not reveal a breach in the epithelial basement membrane. Though the presence of epithelial islands could either be a sectioning artifact or a carcinomatous feature, the tendency to overlook the diagnosis may worsen the prognosis of the patient. Hence, we subjected the processed specimen for serial sectioning. Unexpectedly, the deeper sections revealed a mild breach of epithelium and infiltration of the malignant epithelial cells in the connective tissue area (Figure 4).

The histopathologist then arrived at a diagnosis of early invasive oral squamous cell carcinoma from VC. Typically, all lesions representing a verrucoid pattern are always to be studied with serial sectioning; if the deeper sections are not thoroughly investigated, the diagnosis may alter the prognosis. The conventional treatment of oral squamous cell carcinoma is a wide surgical excision with radiation, while the optimal treatment for VC is surgery alone. The above patient was referred for conventional cancer therapy and is under follow-up monitoring.

## Conclusions

OSMF is considered to be a potentially malignant disease. Encountering an exophytic growth over the palpable bands raised our suspicions for a hidden malignancy. Considering the transformation rate of VC to oral squamous cell carcinoma, additional histologic features were required. Though the initial sections were representative of VC, the deeper sections revealed invasive oral squamous cell carcinoma.

VC is generally considered less aggressive than squamous cell carcinoma with a better prognosis and, therefore, requires a more conservative management strategy than squamous cell carcinoma. Thus, the histopathological distinction is important for proper management. The patient’s entire treatment plan and prognosis would have varied if the serial sections had not revealed the hidden malignancy. Therefore, thorough histopathology is a gold standard diagnostic tool for the proper diagnosis of such entities. 
